# Protective Mechanisms of Quercetin Against Myocardial Ischemia Reperfusion Injury

**DOI:** 10.3389/fphys.2020.00956

**Published:** 2020-07-31

**Authors:** Yu-Min Zhang, Zhen-Ye Zhang, Ru-Xing Wang

**Affiliations:** Department of Cardiology, Wuxi People’s Hospital Affiliated to Nanjing Medical University, Wuxi, China

**Keywords:** quercetin, ischemia/reperfusion injury, oxidative stress, vasodilatation, calcium overload

## Abstract

Quercetin has attracted more attention in recent years due to its protective role against ischemia/reperfusion injury. Quercetin can alleviate oxidative stress injury through the inhibition of NADPH oxidase and xanthine oxidase, blockage of the Fenton reaction, and scavenging of reactive oxygen species. Quercetin can also exert anti-inflammatory and anti-apoptotic effects by reducing the response to inflammatory factors and inhibiting cell apoptosis. Moreover, it can induce vasodilation effects through the inhibition of endothelin-1 receptors, the enhancement of NO stimulation and the activation of the large-conductance calcium-activated potassium channels. Finally, Quercetin can also antagonize the calcium overload. These multifaceted activities of Quercetin make it a potential therapeutic alternative for the treatment of ischemia/reperfusion injury.

## Introduction

Quercetin (Que) is a flavonoid that is commonly found in fruits, vegetables, leaves, and Chinese herbs. It is highly present in daily human aliment, such as onions, apple, red wine and tea, which are taken daily by human beings (Wang et al., 2019). It exerts various biological effects including antioxidant, anticancer, anti-inflammatory, anti-aggregatory, anti-aging effects (Iskender et al., 2017). Que is non-toxic and safe to animals, even at high doses (4000 mg/day), which makes it safe for dietary intake (Jiang et al., 2018). Indeed, Que has been used as a drugstore medicine in certain countries. In 2010, Que supplements were added to Food and Drug Administration’s Generally Recognized as Safe (GRAS) list at the dosage of 500 mg, which was recognized as an effective dosage in many trials (Dabeek and Marra, 2019). In addition, Que has been recently shown to have prominent potentials to ameliorate myocardial damage under ischemia/reperfusion (I/R) condition through various pathways.

Acute myocardial infarction is a seriously growing global health problem due to its high mortality and morbidity rates (Huang et al., 2015). Most of physicians prefer to rapidly restore the coronary blood flow as a first choice when I/R occurs (He et al., 2017). However, myocardial reperfusion itself can cause a serious damage to the myocardium, leading to inflammatory reactions, dysfunction of ion channels, mitochondrial impairment and excessive oxidative stress (Wu et al., 2018), which can consequently lead to cardiac dysfunction. In this review, we discuss the potential protective mechanisms of Que against myocardial I/R injury.

## The Structure Of Quercetin

**FIGURE 1:**
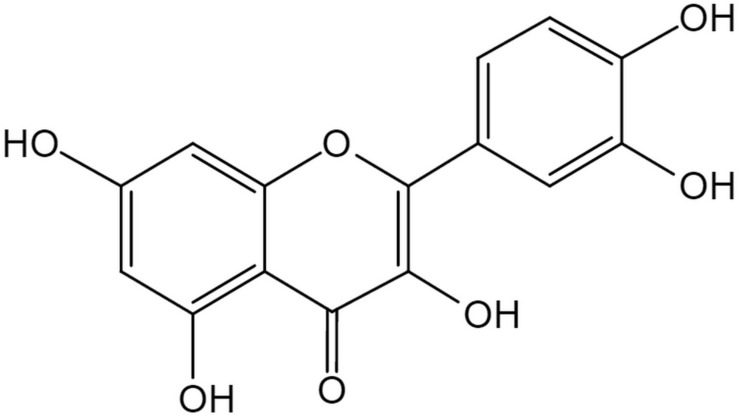
The structure of Quercetin. The flavonoid Quercetin is a 3,30,40,5,7-pentahydroxyflavone with the chemical formula C_15_H_10_O_7_, a member of flavonoids. Quercetin has a strong antioxidant property due to the presence of five hydroxyl groups in the structure and the pyrocatechol-kind of benzene ring makes it a good scavenger of free radicals.

The flavonoid Que is a 3, 30, 40, 5, 7-pentahydroxyflavone with the chemical formula C_15_H_10_O_7_ (Andres et al., 2018). There are currently more than 5000 flavonoid compounds with known different conformations. They exist in most of the plants and play a vital role in vegetative growth, development and flowering. Flavonoids are commonly found in combination with glycosyl to form stable glucosides (Perez-Vizcaino and Fraga, 2018). In general, they are divided into flavones, flavanol, flavanones, flavanonol, anthocyanidins, flavan-3, 4-diols, xanthones, chalcones, and bioflavonoids (Patel et al., 2018). Generally speaking all flavonoid are hydrophobic due to the presence of two benzene rings, and a pyran ring between them, in their structure (Tawani et al., 2017). Que has a strong antioxidant property due to the presence of five hydroxyl groups in the structure. Furthermore, the pyrocatechol-kind of benzene ring makes it a good scavenger of free radicals (Xu et al., 2019). As one of the most popular members in the flavonoids family, Que accounts for 65–75% of our daily intake of flavonoids (Suganthy et al., 2016). In nature, mainly in vegetables and fruits, Que exists in the form of ramifications that bind to glucose and rutinose (Figure 1; Terao, 2017). Thus, Que can be rapidly hydrolyzed by β-glucosidase enzyme once ingested through the digestive tract, which makes it easier for absorption by mucosa of the large intestine, and then transferred to the whole body through the portal circulation (Zhang L. et al., 2018).

## The Biological Value of Que

Que has drawn global scientific attention due to its various and unique biological values. Indeed, it can exert anti-diabetic, anti-tumor and anti-obesity effects (Lu et al., 2017). We will focus on its anti-inflammatory, anti-oxidant and anti-apoptotic effects in detail in the following part.

### Que Anti-diabetic Effects

The 5′-adenosine monophosphate-activated protein kinase (AMPK) signaling is associated with cellular glucose uptake and glucose production (Zou et al., 2018). However, the AMPK signaling is impaired in diabetes. Despite that certain drugs like metformin can regulate the activity of AMPK, they also have some adverse effects like hypoglycemia (Joshi et al., 2019). On the other hand, it showed that Que can activate AMPK signaling and improve insulin resistance in skeletal muscle cells and stimulate insulin secretion (Eitah et al., 2019).

### Que Anti-obesity Effects

Que has been reported to decrease blood glucose, serum lipids, liver triacylglycerol, body weight and liver fat accumulation in the C57BL/6J mouse model on high-fat diet (Que 0.025% w/w) (Jung et al., 2013). Besides, similar effects have been shown *in vitro* that are in line with these *in vivo* findings. Indeed, Que (25 μM) was added to the cell cultures and it reduced the accumulation fatty acids and their metabolites in human Simpson Golabi Behmel Syndrome (SGBS) preadipocytes, probably through the *Enolase 2* gene that is vital for the conversion 2-phosphoglycerate into phosphoenolpyruvate (Leiherer et al., 2016).

### Que Anti-tumor Effects

Que can inhibit the progression of human cancers (i.e., breast cancer, colon cancer), with little or no harm to normal cells (Rauf et al., 2018). Moreover, Que plays a synergistic role to increase treatment sensitivity protecting patients from adverse effects in combination with chemotherapy and radiotherapy (Lei et al., 2017). Que can inhibit the growth of breast cancer through the suppression of the p-AKT/AKT signaling pathway. Besides, Que (50 mg/kg intraperitoneal injection) was shown to restrict distant cancer metastasis through the downregulation of the AKT-mTOR pathway (Jia et al., 2018).

### Quercetin Protective Effects Against Metabolic Syndrome

Metabolic syndrome has been a major health concern due to its relationship with cardiovascular diseases. Defined as patients with dyslipidemia, hyperglycemia, or systemic hypertension. Metabolic syndrome is linked to a 2-fold increase in cardiovascular diseases and a 1.5-fold increase in all-cause mortality (Mottillo et al., 2010). In a clinical trial, a decrease in blood pressure was found after 6 weeks continuous daily intake of Que (150 mg/kg). Meanwhile, a significant decrease in low density lipoprotein was also observed. However, there were no pronounced changes in blood glucose. These effects were more obvious in younger metabolic syndrome patients aged 20–25 years compared with the whole group, and subjects with specific apolipoprotein-E genotypes had differential lipid responses to Que (Amiot et al., 2016).

## The Main Mechanisms of Myocardial I/R Injury

**FIGURE 2:**
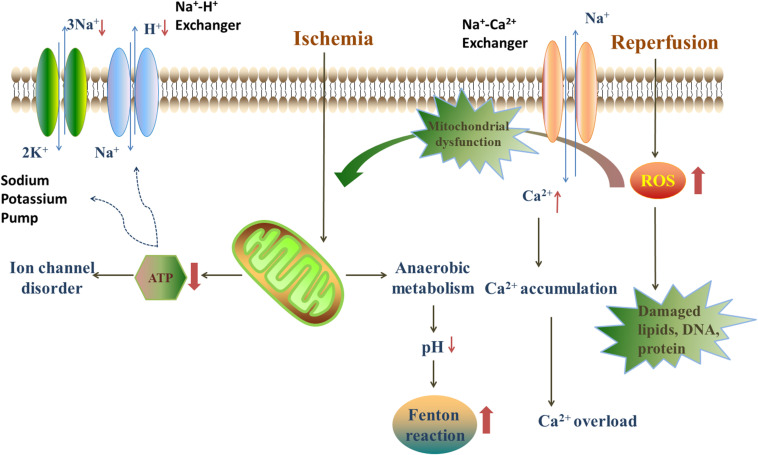
The injury mechanism in ischemia/reperfusion period. During the ischemic period, anaerobic metabolism, and anaerobic glycolysis are promoted, leading to a decrease in oxygen tension, ATP generation and the decline of pH. The lack of ATP leads to the disorder of ion exchanges. The decline of pH provides an acidic environment for Fenton reaction. Blood restoration disturbs the balance of oxidation/anti-oxidation system and quick sodium-calcium exchange. The quick sodium-calcium exchange causes the Ca^2+^ accumulation and impairs the mitochondria. Furthermore, the reperfusion leads to a burst of ROS, mainly deriving from NADPH oxidase, xanthine oxidase and Fenton reaction.

The mechanisms of myocardial I/R injury have been investigated for several decades and several hypotheses have been proposed. Generally, the progression of myocardial I/R injury is divided into four different periods: ischemia, reperfusion, stress, and death. During the ischemic period, the supply of oxygen to the cells decreases, compared with normal conditions, due to the partial or overall blockage of coronary arteries. Thus, anaerobic metabolism and anaerobic glycolysis are promoted under hypoxia, leading to a decrease in oxygen tension and ATP generation. Under these circumstances, the ion exchange in cellular membranes will be in deregulated, and Ca^2+^ start to accumulate in myocardial cells. The increasing Ca^2+^ concentrations will trigger inflammatory reactions, which results in the stimulation of cytokines, and consequently, the death of myocardial cells (Li et al., 2019). In the reperfusion period, restoration of the blood supply brings oxygen to the ischemic myocardium, which leads to a burst in the generation of reactive oxygen species (ROS) that will damage the electron transfer chain in the mitochondria (Lee et al., 2018). Besides, ROS also oxidize lipids, DNA and proteins, and impair the cell structure, which finally lead to cell death (Figure 2; Yu et al., 2017).

## Que Effects Against Oxidative Stress

There is a famous French paradox that the rate of coronary heart disease-related death is low despite high intake of dietary cholesterol and saturated fat (Siasos et al., 2013). This observation could be related to the large consumption of red wine by French people, in which Que is one of the major components that have strong anti-oxidative and vasodilatation effects (Rendig et al., 2001). Myocardial I/R injury induces the excessive production of ROS, which are responsible for oxidative stress-mediated injury in cardiomyocytes (Zhao et al., 2017). Excessive oxidative stress is characterized by the disruption of the balance between oxidation and anti-oxidation systems, with a tendency toward oxidation. Oxidative stress can lead to numerous reactions, such as neutrophil infiltration, stimulation of proteases, and a burst of oxidative intermediates. In addition, oxidative stress raises multiple adverse effects in the body, which are mainly caused by ROS. The latter exists in the body in various forms, such as H_2_O_2_, O_2_^⋅–^, OH^⋅^, ^1^O_2_. H_2_O_2_ is the most stable and the most abundant form present in the human body (Chen et al., 2013). ROS mainly come from the mitochondrial electron transfer chain, NADPH oxidase, myeloperoxidase (MPO), and xanthine oxidase. In the normal physiological state, ROS play important roles as a signaling messenger in various signaling pathways, such as cell proliferation and pressure adaption (Sugamura and Keaney, 2011). In addition, low levels of ROS also play a role in ischemic preconditioning (Becker, 2004), where a burst in ROS production occurs after exposure to an adverse condition, such as ischemia and hypoxia. Excessive ROS then destroy the inherent antioxidant system, impairs myocardial cells and causes a series of problems, such as protein degradation, lipid oxidation and DNA damage, mainly to mitochondrial DNA (Seidlmayer et al., 2015). Besides, ROS can also lead to the induction of mitochondrial permeability transition pore (mPTP) formation, which will increase mitochondrial membrane permeability (Cao et al., 2017). As a result, this may lead to several damages in the plasmalemma, which will further aggravate cell apoptosis. Moreover, ROS can further destroy the cell structure by reacting with membrane phospholipids and inducing lipid peroxides formation (Chouchani et al., 2014). The impairment of electron transfer chain in the mitochondria will in turn lead to more ROS production and mitochondria damage, which is one of the major injury mechanisms of I/R condition (Hinchy et al., 2018).

Along with the I/R injury, excessive ROS may also lead to arrhythmia during the ischemic period, including ventricular fibrillation, premature ventricular contraction, and ventricular tachycardia (Broskova et al., 2013). Therefore, scavenging of ROS can be a potential target to ameliorate myocardial I/R injury.

### Inhibition of NADPH Oxidase

**FIGURE 3:**
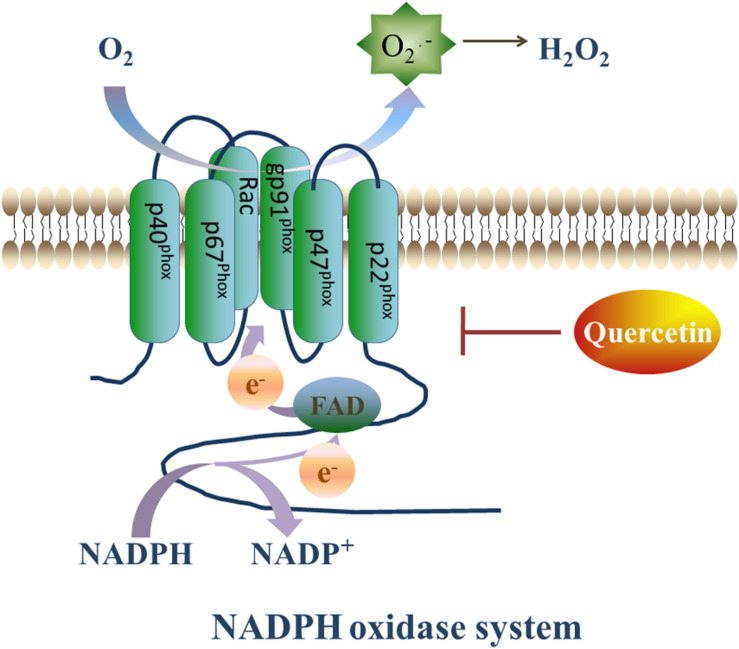
Quercetin’s prohibitive effective to NADPH oxidase. NADPH oxidase plays a vital role in generating ROS. On the setting of NADPH oxidase, NADPH transfers one electron to O_2_, then forms NADP^+^ and O_2_^⋅–^, and O_2_^⋅–^ will transfer into H_2_O_2_ rapidly. However, Que can decrease the expression of Nox2 and ameliorate the oxidative stress.

NADPH oxidase is a complex enzyme that is composed of membranous subunits (gp91-phox, p22-phox), intracytoplasmic subunits (p47-phox, p67-phox, p60-phox) and a small GTP-binding protein Rac. The gp91-phox subunit and its homologs, Nox1, Nox3, Nox4, Nox5, Duox1, and Duox2, are commonly known as the Nox family (Breitenbach et al., 2018). The Nox family expressed in almost all organs, tissues, and cells (Brandes et al., 2014). In the circulatory system, Nox1 is mainly expressed in vascular smooth muscle cells (VSMC), while Nox2 and Nox4 are expressed in endothelial cells, myocardial cells, fibroblasts, as well as VSMCs. On the other hand, Nox3 and Nox5 are almost exclusively expressed in myocardial cells (Figure 3; Drummond and Sobey, 2014).

NADPH oxidase, is the major ROS producer in the body by transferring one electron from NADPH to O_2_, thus forming NADP^+^ and O_2_^⋅–^. The produced O_2_^⋅–^ is rapidly transformed to H_2_O_2_, which is more stable and diffusible (Guichard et al., 2006) and accounts for most of the reactions induced by ROS. Thus, the inhibition of the NADPH oxidase expression to reduce H_2_O_2_ production can mitigate ROS-induced I/R injury.

In myocardial cells, NADPH oxidase can be activated by various agonists and stimulating factors (Anilkumar et al., 2009), such as G-protein-coupled receptors agonists (angiotensin-II, ET-1, and α-adrenoceptor agonists), growth factors [thrombin and vascular endothelial growth factor (VEGF), cytokines tumor necrosis factor (TNF), and transforming growth factor (TGF)], mechanical injury, metabolism related factors (insulin,glucose, and free fatty acid), and ischemia reperfusion. Indeed, Nox2 and Nox4 are activated during myocardial I/R injury. While Nox2 is activated in a complex way that is associated with the post-translational modification of the p47-pox and Rac1 GTPase subunits, the activation of Nox4 is dependent on the post-translational modification of p22-phox only (Martyn et al., 2006).

NADPH oxidase participates in various pathological processes including cardiac hypertrophy, ventricular remodeling, hypertension, and atherosclerosis (Akcaboy et al., 2016). Together with the increase in oxidative stress, the expression and enzymatic activity of the enzyme subunit are enhanced in ischemic tissues.

Of interest, Que has shown a promising protective capacity to attenuate the expression and the enzymatic activity of NADPH oxidase subunits (Sanchez et al., 2006). For instance, in a rabbit model of I/R injury, in which the left anterior descending branch of the coronary artery was ligated for 30 min then released, Nox2 was upregulated both at the RNA and protein levels. However, injection of Que intravenously 5 min before ligation operation attenuated the expression of Nox2. In addition, the expression of the inducible nitric oxide synthase (iNOS) was also suppressed in the Que-treated group, compared with the I/R group without Que treatment (Wan et al., 2009). The iNOS can generate NO, a non-toxic molecule that usually does not induce any damage to the myocardium. However, NO can produce ONOO^–^ when coupled to O_2_^⋅–^, leading to oxidative/nitrative tissue injury. Hence, Que’s prohibitive effect on NADPH oxidase may be a potential target for ameliorating I/R-induced injury.

### Inhibition of Xanthine Oxidase

**FIGURE 4:**
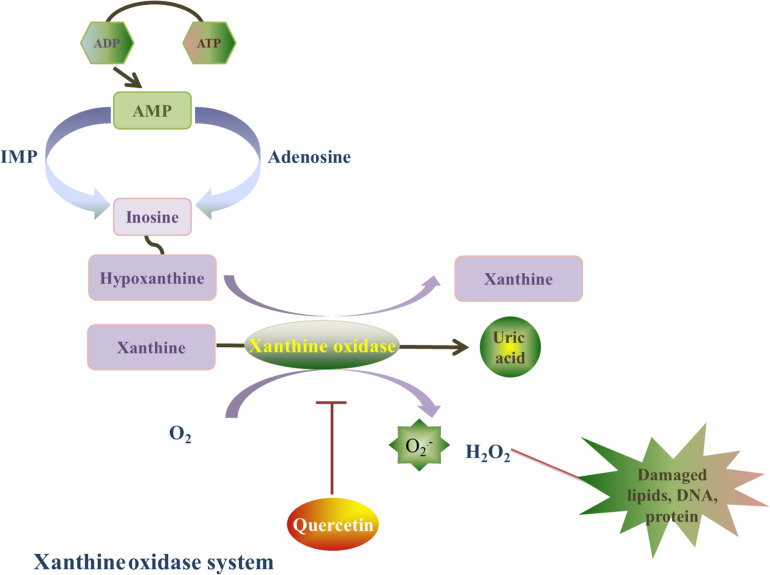
Quercetin’s prohibitive effective to xanthine oxidase. Xanthine oxidase is a homodimer, and it is also a major source of ROS. Xanthine oxidase catalyzes hypoxanthine/xanthine into xanthine/uric acid, and xanthine oxidase itself turns into reduced form. Afterward, xanthine oxidase (reduced form) catalyzes O_2_ into O_2_^⋅–^ and H_2_O_2_. Quercetin can inhibit the O_2_^⋅–^ generation induced by xanthine oxidase, and this effect is associated with the reduced form of xanthine oxidase by a ping-pong mechanism.

Xanthine oxidase is a homodimer that includes two symmetrical monomers, each of which has a C-terminal molybdopterin (Mo) domain containing four redox centers, an N-terminal domain with two iron sulfur centers, and a central flavin adenine dinucleotide (FAD) cofactor (Wilson et al., 2018). Xanthine oxidase (oxidized form) catalyzes hypoxanthine/xanthine into xanthine/uric acid, and xanthine oxidase itself turns into reduced form. Afterward, xanthine oxidase (reduced form) catalyzes O_2_ into O_2_^⋅–^ and H_2_O_2_. The basal level of xanthine oxidase is low, its expression is upregulated under I/R conditions. Xanthine oxidase stimulates redundant ROS production, which can lead to oxidative stress injury (Malik et al., 2018). In addition, xanthine oxidase has been shown to play a role in atrial remolding induced by oxidative stress in alloxan-induced diabetic rabbits (Figure 4; Yang et al., 2018).

Que ameliorates oxidative stress injury caused by xanthine oxidase and reverses myocardial remodeling after myocardial ischemia (Barteková et al., 2015). Indeed, Que can inhibit xanthine oxidase-mediated O_2_^⋅–^ generation, which is related to the reduced form of xanthine oxidase by a ping-pong mechanism (Zhang C. et al., 2018). In a hyperuricemia animal model induced by potassium oxonate (uricase inhibitor) (Huang et al., 2011), a dose-dependent reduction in xanthine oxidase expression and enzymatic activity was detected after Que (100,200, 400 mg/kg) treatment for 7 continuous days. The inhibition of xanthine oxidase by Que was associated led to a decrease in oxidative stress. Hence, the inhibitory effect of Que on xanthine oxidase warrant further investigation.

### Activation of the Selenoproteins

Selenium, a kind of trace element, is not only an actual antioxidant on its own but also an integral component of selenoproteins. Among various kinds of selenoproteins, glutathione peroxidases (GPx), thioredoxin reductases (TrxR), and methionine sulfoxide reductase 2 (MsrB) are known as redox-active selenoenzymes, which possess a selenocysteine in their active site (Steinbrenner et al., 2016). By reacting with reduced glutathione, GPx isoenzymes can reduce H_2_O_2_, organic hydroperoxides, and (only GPx4) phospholipid hydroperoxides. TrxR also reduces various kinds of substrates, such as oxidized thioredoxins, H_2_O_2_ and organic hydroperoxides and MsrB reduces free and protein-bound methionine sulfoxide to methionine. Other selenoproteins, which contain a conserved CXXU motif corresponding to the CXXC motif of thioredoxins, are acting as oxido-reductases. They include selenoproteins H, M, O, V, W, and T. Interestingly, Selenoproteins T has been reported to induce post-conditioning myocardial protection by reducing I/R injury through decreasing infarct size and improving post-ischemic cardiac function through the control of various signaling effectors of apoptosis and oxidative stress (Rocca et al., 2018). Alteration of selenoproteins T is also associated with, such as congestive heart failure, coronary diseases, impaired cardiac structure and function (Rocca et al., 2019).

Que can restore the inherent anti-oxidative system and ameliorate I/R injury, and it may be linked to selenoproteins (Arkat et al., 2016). After gavage administration of Que (50 mg/kg) for 8 weeks, TrxR2 increased in Que group compared with Control group. But GPx was found no difference between Que group and Control group. It may be linked to the peroxiredoxin-3, a mitochondrial antioxidant. Peroxiredoxin-3 plays an important role in alleviating cellular stress, and it can be upregulated by Que.

### Inhibition of the Fenton Reaction

Fenton reaction is a strong redox reaction that has been widely used in industry. It was discovered and named by the chemist Fenton HJ (Thomas et al., 2009). In this reaction, a Fe^2+^/Cu^1+^ metal iron transfers an electron to H_2_O_2_, which produces Fe^3+^/Cu^2+^, OH^–^ and OH⋅ (step 1). It has been demonstrated that OH⋅ has a very strong oxidizability in acidic solutions, which is the second after fluorine (Babenkova et al., 2018). In addition, H_2_O_2_ can restore Fe^2+^/Cu^1+^ from Fe^3+^/Cu^2+^, which produces HOO^⋅^ and H^+^ (step 2). Fe^2+^/Cu^1+^ can be oxidized by O_2_ again, which forms O_2_^⋅–^ (step 3). Anaerobic glycolysis in the myocardium is enhanced under ischemia/hypoxic conditions, which provides an acid environment for the Fenton reaction to occur. In addition to the production of strong oxidants, Fenton reaction also stimulates the generation of O_2_^⋅–^.

Que can combine with the Fe^2+^/Cu^1+^ as copper (II)-Que complex and Cu(I)-Que complex, which significantly inhibit the Fenton reaction induced by Fe^2+^/Cu^1+^ and reduces OH⋅ formation, as confirmed by Electron Paramagnetic Resonance (EPR)-spin trapping experiments (Jomova et al., 2017). Interestingly, the Fe^2+^-Que complex showed a stronger radical scavenging capacity, compared with Que alone.

Fe^2+^/Cu^1+^+ H_2_O_2_ → Fe^3+^/Cu^2+^+ OH^–^+OH⋅ step 1

H_2_O_2_ + Fe^3+^/Cu^2+^ → Fe^2+^/Cu^1+^ + HOO^⋅^+H^+^ step 2

O_2_ + Fe^2+^/Cu^1+^ → Fe^3+^/Cu^2+^ + O_2_^⋅–^ step 3

### ROS Scavenging Activity of Que

Que is well-known for its strong ROS scavenging activity, which can prevent oxidative stress-mediated injury of the myocardium under I/R conditions by preventing the accumulation of ROS and the progression of redox reactions. Nevertheless, ROS scavenging can also increase the bioavailability of NO and the restoration of endothelial function after I/R injury (Benito et al., 2002). Que has been shown to scavenge ROS through both direct and indirect mechanisms. The direct scavenging of ROS is mainly related to the ability of flavonoids to react with free radicals, such as O_2_^⋅–^, HO., NO., alkoxyl, and peroxyl radicals. This may occur briefly when hydroxyl bound to benzene ring transfers a hydrogen atom or an electron to the free radical, which will result in the formation of more stable molecules (Sarkar et al., 2012). Consequently, a more stable quinone structure is formed when the phenoxyl radical of Que reacts with a free radical.

On the other hand, Que can scavenge ROS indirectly by restoring the inherent anti-oxidant systems of the body, which include enzyme-dependent and enzyme-independent systems. The enzyme-independent anti-oxidant system includes vitamin C, vitamin E, and glutathione (GSH) (Liu et al., 2012), which act as first line defense against oxidative stress-mediated injury by capturing ROS and inhibiting ROS-induced myocardial injury. However, this system could be consumed if the oxidative stress overwhelms the anti-oxidant system. The enzyme-dependent anti-oxidant system, on the other hand, includes superoxide dismutase (SOD), catalase (CAT), glutathione peroxidase (GHS-Px), and peroxidase (POD). These enzymes work in synergy and play an important role in the anti-oxidant system. SOD is a metalloenzyme that possesses an anti-oxidant capacity by catalyzing the transformation of the radical O_2_^⋅–^ into O_2_ and H_2_O_2_; the latter is then discomposed by CAT. SOD is divided into Cu/Zn-SOD, Mn-SOD, and Fe-SOD. Cu/Zn-SOD is the most commonly expressed SOD enzyme in the human body (Son and Elliott, 2014). GHS-Px is an important peroxide clastic enzyme that transfers GSH into oxidized glutathione (GSSG), while POD can transform the toxic peroxide into nontoxic hydroxy (Ekoue et al., 2017).

It was reported that non-enzymatic anti-oxidants, such as vitamin C, vitamin E, and GSH, decrease after I/R injury, which indicates a depletion of the anti-oxidant system. Nevertheless, Que can eliminate ROS and restore the function of the intrinsic anti-oxidant system. In this regard, cardiomyocytes, isolated from 2-days old neonatal Sprague-Dawley rats, were incubated under hypoxia (anoxia,5% CO_2_ and 95% N_2_) for 4 h, then transferred to atmospheric oxygen tension (21% O_2_, 5% CO_2_, and 74% N_2_) for 2 h (Tang et al., 2013). In this anoxia/re-oxygenation injury model, an increase in creatine phosphokinase and lactate dehydrogenase (LDH) was detected, which indicates impairment of myocardial cells. However, this was accompanied with an increase in malondialdehyde (MDA), the end product of lipid metabolism, and a decrease in SOD and GSH-Px, which indicate that the intrinsic anti-oxidant system is consumed and the injury is mainly caused by oxidative stress. Interestingly, Que (10,20,40, and 80 μM) treatment increased SOD and GSH-Px in this model, but also recovered the function of the mitochondria. Mitochondrial membrane potential (MMP) is a marker that reflects the function of the mitochondria. In the I/R group without Que treatment, there was a decrease in MMP, which indicates that the efficacy of the mitochondria is impaired. However, Que treatment attenuated the decrease in MMP, which suggest the restoration of mitochondrial function.

It has been reported that the mechanism of these changes is associated with PKCε, a member of the PKC family, which is involved in various physiological processes including mitosis and the regulation of transcription and cellular survival under stress conditions (Maurya and Vinayak, 2015). In the I/R rats cardiomyocytes treated with Que (10,20,40,80 μM), the upregulated expression of PKCε was suppressed by εV1-2, a selective PKCε inhibitor, which suggest that the protective effects induced by Que are mediated by PKCε. However, the other member of PKC family like PKCα, PKCβ and PKCη were found decrease after treated with Que (Tang et al., 2013). It may be associated with ROS, which take part in the signaling pathways of PKC and result in the activation of the total PKC. The ROS scavenging activity of Que leads to the decrease of ROS then inhibit the activity of PKCα, PKCβ and PKCη.

## The Anti-Inflammatory and Anti-Apoptotic Effects of Que

I/R injury intensifies acute inflammatory reactions in the myocardium by neutrophil granulocytes, which are vital leukocytes that account for most of the exudation in I/R injury. Indeed, diffusion of neutrophils from the blood vessel through chemotactic effects can further impair the injured tissue (Rohrbach et al., 2015). In myocardial tissues under I/R injury, the damaged endothelial cells induce leukotriene B4, intercellular adhesion molecules (ICAM), vascular cell adhesion molecules (VCAM) and selectins, which play a role in the chemo-attraction, detainment, and conglutination of neutrophils to the vessel wall. In I/R period, leukocytes can impair the function of endotheliocytes and reduce the relaxation capacity of coronary arteries (Akhlaghi and Bandy, 2009), but also they promote the coagulation and proinflammatory effects, which further exacerbate the mechanical obstruction of capillaries. The incomplete perfusion of coronary arteries and the decrease of blood flow are the main causes of neutrophil’s capture and conglutination to vascular walls (Li J. et al., 2012). The firm attachment of leukocytes to dysfunctional endotheliocytes plays an important role in the inflammatory reaction. The latter is accompanied with myocardial apoptosis, which initiates at the beginning of ischemia, expands by reperfusion, and partly induces the death of all cells. The increase in plasmalemma’s permeability leads to the release of cytochrome C, which further exacerbate myocardial apoptosis (Barteková et al., 2015).

Que has been shown to ameliorate inflammation and apoptosis in the myocardium both *in vitro* and *in vivo*. An I/R injury model was established by the ligation of left coronary artery, which was followed by Que (2,10, and 20 mg/kg per os) and diltiazem (15 mg/kg per os) administration for 5 days. Interestingly, Que treatment inhibited the expression of TNF-α, IL-6, and IL-1β in the serum and myocardial cells, compared with the control I/R group and the diltiazem-treated I/R group. TNF-α, IL-6, and IL-1β are important biomarkers of inflammatory reactions. In line with the decrease in inflammatory biomarkers, creatine kinase (CK), and LDH were downregulated, compared with the control and the diltiazem-treated I/R groups, which indicates that Que treatment after I/R can alleviate myocardial inflammation and apoptosis. In addition, the electrocardiograms in the I/R group demonstrated an elevation in the ST-segments and a reduction in the R-amplitude, which indicates that the I/R model was successfully produced. Interestingly, Que treatment ameliorated these alterations, but also restored the infarct sizes, which were detected by 2,3,5-triphenyltetrazolium chloride (TTC) staining. In addition, the vitality of the coronary artery was enhanced, as indicated by the increase in coronary flow in I/R rats treated with Que group, compared with the control I/R group. Furthermore, myocardial contractility was also partly reinforced after administration of Que. All these results indicate that Que can mitigate inflammation and restore the myocardial function after I/R injury.

The mechanism of these changes could be related to the high mobility group box-1 (HMGB1) signaling. HMGB1 is a high conservative nucleoprotein that is involved in the stabilization of DNA and enhancement of transcription (Tang et al., 2017). However, recent studies have shown that HMGB1 plays an important role as a mediator of early inflammation. Indeed, the injured myocardium can produce HMGB1 passively under ischemic circumstance, or actively by stimulation with immunocytes, such as macrophagocytes, mononuclear leucocytes and dendritic cells, which can further aggravate the inflammatory reaction. Accumulating evidence have shown that activated HMGB1 can upregulate the expression of extracellular regulated protein kinases (ERK)1/2 and NF-KB by interacting with Toll-like receptors (TLRs). The upregulation of NF-KB further increases oxidative stress and apoptosis, leading to a reduction in the vasodilation and anti-oxidative capacity of the artery. Interestingly, the overexpression of HMGB1 was also detected in the I/R rat model, along with an increase in TLR4 and NF-KB expression. On the other hand, Que treatment decreased the expression of inflammatory biomarkers, such as TNF-α, IL-1β, and IL-6, compared with non-treated I/R group, but also it attenuated inflammation and apoptosis induced by I/R injury via HMGB1/TLR4/NF-KB signaling.

## The Vasodilatory Effects of Que

During the ischemic period, the coronary arteries are usually blocked by emboli after plaque disruption. Although blood flow can be re-established rapidly by interventional therapies and medications, multiple findings support that vasoconstriction-associated G protein-coupled receptors are upregulated in VSMC after I/R injury (Skovsted et al., 2014). The mechanisms of vasoconstriction and vasodilatation are mainly classified as endothelium dependent or VSMC mediated mechanisms, wherein Que can promote the vasodilatory effects through both mechanisms.

### Vasodilatory Effects Against Endothelin

Endothelin is one of the most potent vasoconstrictors that are mainly distributed in blood vessel endothelium, but also in multiple other tissues and cell types (Kaoukis et al., 2013). It is an important factor in mediating cardiovascular functions, in addition to stabilizing basic vascular tension and maintaining cardiovascular homeostasis. It is a polypeptide that is composed of 21 amino acids, with two disulfide bonds in the N-terminus that connect cysteines at the 1–15 and 3–11 sites to the hydrophobic amino acid in the C-terminus. The N-terminal structure determines the appetency of endothelin to its receptor, while the C-terminal structure determines the position that bind to the receptor (Dammanahalli and Sun, 2008). There are three types of endothelin: ET-1, ET-2, and ET-3. ET-1 plays a major role in the cardiovascular system homeostasis through the GPCRs ET_A_ and ET_B_ receptors (Lichtenauer et al., 2017). While the ET_A_ receptors are commonly expressed in VSMCs and induce vasoconstriction, the ET_B_ receptors are mainly expressed in endotheliocytes and are associated with vasodilatation. However, ET_B_ has been described to function as a vasoconstrictor in VSMCs (Iglarz et al., 2015). Indeed, the expression of the ET_B_ receptor and its mediated vasoconstriction were shown to be significantly increased in coronary arteries after I/R injury (Wackenfors et al., 2004), and were associated with the damage induced after I/R injury.

Administration of Que can ameliorate vasoconstriction caused by ET-1. *In vitro* administration of Que to Chinese hamster ovary cells, expressing the calcium sensitive apo-aequorin protein (CHO-AEQ) and transfected with human ET_A_ or ET_B_ receptors, partly impeded intracellular Ca^2+^ signaling. Interestingly, a decrease in the G protein-coupled receptor ET_A_ was also observed (Bahem et al., 2015). Similar results were also observed in 12 healthy humans daily administrated with 200 mg of Que for 7 consecutive days, where a significant decrease in urinary ET-1 concentrations was observed (Loke et al., 2008). In pig isolated coronary artery, Que caused concentration dependent inhibition of endothelin-1-induced contractions and it was slow at the beginning and required 30 min to achieve equilibrium (Suri et al., 2010).

### NO-Mediated Vasodilatory Effects of Que

NO is an important mediator of vasodilation, but also a shield of the nervous system and an anti-inflammatory factor (Simmonds et al., 2014). During I/R, uncoupling of the NO synthase (NOS) reduces the generation of NO and promotes the generation of O_2_^⋅–^ (Silberman et al., 2010). These effects can be reversed after the administration of Que, which augments the production of NO through the activation of endothelial NOS (eNOS) (Romero et al., 2009). Besides, Que can scavenge O_2_^⋅–^, and thus, reduce the consumption of NO.

Treatment of bovine aortic endothelial cells (BAECs) with Que induced the phosphorylation of eNOS at Ser1179 in a time- and dose-dependent manner, and this effect was abolished by the PKA inhibitor H89. Que treatment also enhanced the production of NO, which was inhibited by the eNOS inhibitor, L-NAME. Besides, the authors observed a vasodilatory effect in aortic rings after treatment with Que (10,25,50 μM) (Li P. G. et al., 2012).

### Vasodilatory Effects of Que on VSMCs

L-type voltage-gated Ca^2+^ channels and voltage-gated K^+^ channels are commonly expressed in VSMCs and are associated with the coronary artery tone. L-type voltage-gated Ca^2+^ channels, known as long-lasting channels, mainly exist in VSMCs and play a major role in excitation-contraction coupling. On the other hand, voltage-gated K^+^ channels play a vital role in maintaining membrane potential and in regulating the vascular tone. In normal coronary smooth muscle cells, large conductance Ca^2+^-activated K^+^ channel (BK channels) account for 65% of potassium current and are closely associated with vasodilatation.

Recent studies have shown that Que can inhibit L-type voltage-gated Ca^2+^ channels and enhance BK channels. Vascular tension was recorded using wire myograph showed that Que can induce vasodilatation in vascular rings isolated from Male Sprague-Dawley rats by decreasing the vasoconstrictive effects induced by KCl, reducing cytoplasmic Ca^2+^ concentrations, and suppressing the inward Ca^2+^ currents through L-type voltage-gated Ca^2+^ channels (Hou et al., 2014).

Similarly, Que (3,10,30 μM) can lead to a vasodilatory effect by increasing the opening rate of BK channels in coronary smooth muscle cells. This effect caused by Que is mainly mediated through its H_2_O_2_ generating capacity under certain condition (Duarte et al., 2004). In this regard, Que behaves as a pro-oxidant producing ROS, which are involved in signal transduction in coronary arteries, whereby ROS can activate BK channels in coronary smooth muscle cells (Cogolludo et al., 2007).

## Protective Effects Against Calcium Overload

The intercellular environment is an important place for the exchange of substances between cells and their outer environment. Intracellular ionic homeostasis provides a stable environment for various physiological reactions. The calcium pump (Ca^2+^-ATP enzyme) and the sodium-potassium pump (Na^+^-K^+^-ATP enzyme) play an important part in maintaining the balance of ion exchange (Stein, 2002). However, under ischemic conditions, the ATP content of the cell decreases, which can lead to the depression of the sodium-potassium pump, and consequently, to a decrease in intracellular sodium content. In the reperfusion state, the calcium pump in cardiocytes is activated after supplementation with O_2_ and nutrient. This results a quick exchange of Ca^2+^ and Na^+^, and thus, leads to high accumulations of Ca^2+^ (calcium overload) (Tu et al., 2019). Calcium overload can impair the process of oxidative phosphorylation in the mitochondria and decreases ATP content, which reduces the membrane potential and lead to serious irreversible damage to the cell.

Que can relieve cellular impairment caused by calcium overload and can decrease Ca^2+^-dependent cell death when added (1–30 μM) to H9C2 cardiomyocyte 30 mins before application of H_2_O_2_-induced oxidative stress (Guo et al., 2018). Que also inhibited cell death caused by calcium overload by A23187, a free calcium ionophore that can lead to calcium overload, however, without affecting intracellular Ca^2+^ concentrations (Sakanashi et al., 2008).

In spite of these pleasant biological values have confirmed above, Que’s clinical use was still limited due to its poor water solubility and instability in physiological media, leading to poor bioavailability. In a randomized, double-blinded placebo-controlled crossover trial, blood pressure and serum lipids were found no difference between placebo group and Que group after administration of Que (190 mg/day) for 8 weeks (Burak et al., 2019). In another trail, subjects received Que 3-glucoside (160 mg/day) for periods of 4 weeks, however, plasma advanced glycation end products (AGEs) and plasma lipids were found no obvious difference between Que group and control group expect for methylglyoxal (Eynde et al., 2018). The scavenging effect of Que on methylglyoxal, may potentially form a new treatment strategy for diseases in which methylglyoxal plays a pivotal role. A novel soluplus polymeric micelle, used as quercetin-loaded polymeric micelles, was found could improve the absorption *in vitro* and *in vivo* (Chen et al., 2018). A 2.19-fold longer half-life and a relative oral bioavailability of 286% was also observed compared to free Que (Dian et al., 2014). Moreover, Que derivatives were also developed to overcome its limitation, such as 2-(3,4-Diacetoxyphenyl)-4-oxo-4H-chromene-3,5,7-triyl triacetate (Q2), 2-(2,2-Diphenylbenzo[d][1,3]dioxol-5-yl)-3,5,7-trihydroxy-4H-chromen-4-one (Q3), 2-(2,2-Diphenylbenzo[d][1,3]dioxol-5-yl)-4-oxo-4H-chromene-3,5,7-triyl triacetate (Q4), 2-(3,4-Diethoxyphenyl)-3,5,7-triethoxy-4H-chromen-4-one (Q5), 3,5,7-Tris(benzyloxy)-2-(3,4-bis(benzyloxy)phenyl)-4H-chromen-4-one (Q6). Among these derivatives, Q2 and Q5 have better bioavailability and stability than other derivatives. It was found that Q2 has a better anti-obesity effect and remission on metabolic disorders with respect to Que (Nettore et al., 2019). The evaluation of the bioavailability of Q5 has reached 19% at the end of the experiment compared to Que (11%) *in vitro*. A better coronary dilation effect was also found *in vivo*. Through the replacement of hydroxyl groups with ethyl groups, Q5 possesses a better biological efficacy (Grande et al., 2016). In near future, more strategies will be raised which make a promising carrier candidate with efficient delivery of Que for therapeutic treatment.

## Conclusion

I/R injury can lead to many complications as a result of oxidative stress, calcium overload and mitochondrial injury. As we discussed in this review, various findings suggest that Que holds a great potential to ameliorate I/R injury, mainly through anti-oxidative effects. However, more clinical studies are required to elucidate the elusive effective concentrations of Que. Nevertheless, through daily intake, Que has enormous potentials for protecting against I/R injury.

## Author Contributions

Y-MZ and Z-YZ wrote the initial drafts. R-XW revised the review and finalized the last version of the manuscript. All authors contributed to the article and approved the submitted version.

## Conflict of Interest

The authors declare that the research was conducted in the absence of any commercial or financial relationships that could be construed as a potential conflict of interest.

## Abbreviations

AMPK: 5′-adenosine monophosphate-activated protein kinase
BAECs: bovine aortic endothelial cells
BK channels: large-conductance calcium-activated potassium channels
CAT: catalase
eNOS: endothelium nitric oxide synthase
ERK: extracellular regulated protein kinases
FAD: flavin adenine dinucleotide
GHS-Px: glutathione peroxidase
GSH: glutathione
GSSG: glutathione
HMGB1: high mobility group box-1
I/R: ischemia/reperfusion
ICAM: intercellular adhesion molecule
iNOS: inducible nitric oxide synthase
ip: intraperitoneal injection
LDH: lactate dehydrogenase
MDA: malondialdehyde
MMP: mitochondrial membrane potential
MPO: myeloperoxidase
mPTP: mitochondrial permeability transition pore
NOS: NO synthase
po, per os POD: peroxidase
Que: quercetin
ROS: reactive oxygen species
SOD: superoxide dismutase
TGF: transforming growth factor
TLR: Toll-like receptors
TNF: tumor necrosis factor
TTC: 2,3,5-triphenyltetrazolium chloride
VCAM: vascular cell adhesion molecules
VEGF: vascular endothelial growth factor
VSMC: vascular smooth muscle cells.
